# Current antibody-based immunoassay algorithm failed to confirm three late-stage AIDS cases in China: case report

**DOI:** 10.1186/1743-422X-7-58

**Published:** 2010-03-15

**Authors:** Yan Li, Jin-Kou Zhao, Ming Wang, Zhi-Gang Han, Wei-Ping Cai, Bo-Jian Zheng, Hui-Fang Xu

**Affiliations:** 1 Guangzhou Center for Disease Control and Prevention, Guangdong, PR China; 2 School of Public Health, Sun Yat-sen University, Guangdong, PR China; 3 Bill & Melinda Gates Foundation, Beijing Representative Office, Beijing, PR China; 4 Guangzhou No 8 people's hospital, Guangdong, PR China; 5 The University of Hongkong, Hongkong

## Abstract

**Background:**

Immunoassays composed of screening and confirmation are the established algorithm to confirm HIV infection in China, with a Western blot result as the final diagnosis.

**Case presentation:**

In this report, three late-stage AIDS patients were initially tested HIV antibody positive using multiple screening kits, but tested indeterminate using Western blot. HIV infection diagnosis was confirmed based on nucleic acid assays, clinic manifestations and epidemiological history. Case A was identified positive at 30 months, using Western blot, Case B at 8 months, and case C remained indeterminate until he died of Kaposi's sarcoma 4 months after HAART.

**Conclusion:**

The report indicates that current antibody-based testing algorithms may miss late-stage AIDS patients and therefore miss the opportunity for preventing these cases from further transmission. The report also implies that viral load assays is not easy to be universely applicated in developing country like China although it is helpful in diagnosing complicated cases of HIV infection, so the counselling before and after testing is imperative to the diagnosis of HIV infection and risk behavior survey on the examinee should be as detailed as possible.

## Background

For the diagnosis of HIV infection in China, most diagnostic laboratories use enzyme-linked immunosorbent assay (ELISA) tests for HIV antibody screening; further detection of a positive screened sample is usually carried out by using Western blot, which confirms the presence of anti-HIV antibodies[1]. Usually, the high sensitivity and specificity of currently used licensed screening and confirmation reagents can ensure satisfactory results, including relatively low frequency of false-negative and false-positive results. However, in some instances, cases cannot be diagnosed accurately and in a timely manner if only the antibody was tested, such as during the seroconversion "window period", the lack of specific humoral immune response in late-stage AIDS resulting from impaired antibody production, the infection of distinct HIV variants, etc[2,3]. Here we report three late-stage AIDS patients, who were identified as positive on screening tests, but persistently indeterminate on the Western blot assays. The final diagnosis of HIV infection was based on viral load assay, clinical manifestation, and epidemiological information.

## Case presentation

### Case A

A 33-year-old housewife was hospitalized in several different hospitals from March to June in 2005 for the following symptoms: persistent diarrhea, wasting, serious throat aches, difficulty in deglutition, coughing. The physical examination identified cervical fungal infection, oral ulcers, and oral candidiasis. Chest X-ray detected bilateral pulmonary lobular pneumonia. In her self-description, there was no history of drug abuse, premarital sex or extramural risk sex behavior, history of commercial blood or plasma collection, operation history and transfusion of blood/blood products. Condoms were never used in her marriage. When her husband was diagnosed with AIDS in June 2005, she accepted an HIV antibody test, with two rapid test (PA) positive reactions and one third-generation ELISA negative reaction, but an HIV-1 Western blot indeterminate result(p24, Figure 1). At the same time, the CD4 cell count was only 17/μL (Supplementary table 1). After half a year of therapy using antibiotics and anti-epiphyte, the aforementioned symptoms were not alleviated, and the patient was transferred to another hospital in January 2006. In the latter hospital, she was tested for HIV again, with results indicating negative status on screening tests and an indeterminate result on the Western blot (p24, Figure 1). AncillarDiagnostic examinations found the following results: 106 copies/mL HIV viral load; 7/μL CD4 cell; CRF01_AE HIV sub-type; normal liver function; "++" urine WBC; negative results for cytomegalovirus (CMV), herpes, HBV, HCV, and syphilis (Supplementary table 1). After informed consent, the patient initiated HAART in February 2006, with the regime being "Stocrin+Stavudine+Lamivudine" The patient was followed up for 30 months after HAART medication, with results indicating that not only did the viral load decrease to lower than the detectable limit; but CD4 cell count gradually increased and reached 323/μL, the HIV antibody re-emerged in June 2008 and WB tested positive (gp160 gp120p24p17, Supplementary table 1). The clinical conditions of the patient also improved greatly, as illustrated by Supplementary table 1.

**Figure 1:**
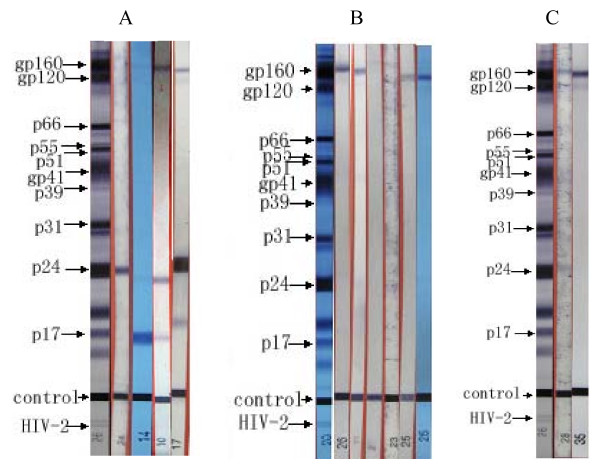
**A: Western blot results for Case A**. Strip 26: positive control; strip 24: p24 (January 19, 2006); strip 14: gp160p24p17 (May 18, 2006); strip 10: gp160p24p17 (December 7, 2006); strip17: gp160 gp120p24p17 (June 5, 2008). **B: Western blot results for Case B**. Strip 20: positive control; strip 26: gp160 gp120 (January 8, 2009); strip 11: gp160 gp120 gp41 (January 22, 2009); strip 27:gp160 (March 30, 2009); strip 27: gp160 (April 30, 2009); strip 25:gp160 gp120 (May 27, 2009); strip 26: gp160 gp120p24 (August17, 2009), the brand was very weak, but allowing the confirmation of HIV infection. **C: Western blot results for Case C**. Strip 26: positive control; strip 28: gp160p24 (may 7, 2009); strip36:gp160 gp120 (August 13, 2009)

### Case B

In January 2009, a 50-year-old female was hospitalized for "a mass in the cervical anterior and a fever that lasted for 17 days." The admission diagnosis was AIDS(C3), oral candidiasis, disseminated penicilliosis, and chronic superficial gastritis. The patient initiated HAART in January 2009, with the regime being "Stavudine+ Lamivudine+Nevirapine" and she was discharged from the hospital after 48 days. During the hospitalization period, HIV antibody examinations indicated strong positives on two third-generation ELISAs (Supplementary table 1), indeterminate on Western blot (gp160 gp120, Figure 1); and CD4 cell count was only 4/μL. In March 2009, she was hospitalized again for "particles trapped in the eyes, dim eyesight and blurred vision for two weeks." She stayed in the hospital for 35 days and the diagnosis at time of discharge was AIDS (C3) combined with a CMV infection. A diagnostic examination found the following results 5 × 103 copies/mL HIV viral load; 11/μL CD4 cell count; CRF01_AE HIV sub-type; positive anti-HBs; 6.42 × 105 copies/mLHCMV-DNA; Chest X-ray detected bilateral pneumonia; floaters eyes; and negative results for herpes, HCV, and syphilis. After 8 months of follow-up, the HIV antibody re-emerged in August 2009 and the WB tested positive result (gp160 gp120p24, Figure 1), along with a CD4 cell count increased from 4/μL to 63/μL.

### Case C

In October of 2008, a 37-year-old man sought medical care for "herpes in the neck". Laboratory results showed that cerebrospinal fluid tested positive for specific IgM antibodies of the herpes simplex viruses, both types1 and 2 (HSV I/II). The patient was discharged from the hospital after symptoms being alleviated. In May 2009, he was hospitalized again for "a rash covering his whole body, continuous fever, coughing, difficulty breathing, and symptoms similar to those of leukoplakia". Further epidemiologic investigation of the patient's sexual history disclosed evidence of premarital sex or extramural risk sex behavior since 2005, with about 50 female and 15 male sex partners, and condoms were seldom used while engaging in sexual activity. HIV antibodies examination indicated strong positives on two third-generation ELISAs and indeterminate on the Western blot (gp160p24, Figure 1). A diagnostic examination found the following results: 106 copies/mL HIV viral load; 35/μL CD4 cell count; CRF01_AE HIV sub-type; positive results for herpes as well as with Kaposi's sarcoma; negative results for HBV, HCV, and syphilis. After informed consent, the patient initiated HAART in May 2009, with a regime being "Kaletra+Stavudine+Lamivudine". Follow-up with the patient continued for 4 months from the commencement of HAART, neither positive HIV antibody nor increase in CD4 cells was found (Supplementary table 1). He died of Kaposi's sarcoma on September 28, 2009.

## Discussion

In this report, complementary and clinical examinations detected low CD4 levels, high viral loads, and two or more kinds of opportunistic infection in all three patients though they were persistently indeterminate on the Western blot assays. The discordant clinical and serological results suggest that there may be an immunological deficiency that prevents the formation of HIV-1 specific antibodies. For cases A and B, CD4 cell count and antibodies gradually increased after HAART, and HIV antibody level was high enough to meet positive test criteria at the end of follow-up. The most plausible explanation is that specific HIV antibodies may have been lost in the end-stage of AIDS and were not sufficient in meeting positive test criteria; the re-emerging of specific antibodies at the end of follow-up may have resulted from the reestablishment of immunity by HAART. For case C, the patient died of Kaposi's sarcoma 4 months after HAART for the failure of reestablishment of immunity.

Apart from the usual low level antibody, the false negative of a diagnosis of HIV infection may also be due to some patients being infected with a very rare or unusual strain of HIV (e.g., HIV-2, or HIV-1 group O)[4]. However, the further testing confirmed that all of the three patients were infected by HIV-1 subtype CRF01_AE. This is in keeping with the local epidemiology of HIV-1 of Guangzhou, where the vast majority of newly diagnosed HIV infections are known to be HIV subtypes BC (51.10%), CRF01_AE (36.9%), and B (10.5%), C (5.3%) [5].

Western blot assays using whole viral lysate antigens have been traditionally considered the "gold standard" for confirming HIV infection[6]. However, several earlier studies have demonstrated the unreliability of this particular assay [7-9]. In the three aforementioned cases, the majority of samples collected during the follow-up periods indicated positive results after screening tests, alleging that HIV antibodies were present; yet, after using the Western blot assay to confirm HIV status, results came out as indeterminate in each of the three cases. However, the Joint United Nations Programme on HIV/AIDS and World Health Organization has recommend three testing strategies involving the use of one to three enzyme-linked immunosorbent assays (ELISA) and/or simple/rapid assays for alternative HIV confirmation. This report is in line with the previous studies [10-12] showed that the alternative strategies may function as well as even better than the current algorithm (ELISA/Western blot) with improved sensitivity, more flexibility and lower cost. Furthermore, there was a lower frequency of discordant or indeterminate results that require follow-up testing, and the accurate diagnosis not only allows patients who need HAART to timely treatment, but also can prevent second-generation transmission.

Moreover, although many studies [13-15] including this report have showed that viral load detection is helpful in the detection and diagnosis of HIV infection, especially when diagnosing complicated and difficult cases of HIV infection, it is not easy to be universely applicated in developing country like China, as the cost is relatively high, and requisite equipment and a proper working environment may be difficult to attain in some instances. So this report also support that the counselling before and after testing is imperative to the diagnosis of HIV infection and risk behavior survey on the eaxminee should be as detailed as possible, the final diagnosis must be based on the laboratory testing results and epidemiological information.

## Consent

Written informed consent was obtained from the patient for publication of this case report. A copy of the written consent is available for review by the Editor-in-Chief of this journal.

## Competing interests

The authors declare that they have no competing interests.

## Authors' contributions

YL carried out the follow-up and the variable testing. JZ contributed to the interpretation of data and critically revised the manuscript. MW and BZ contributed to revising the manuscript. ZH and CW participated in acquisition of data and coordination of participants. HX conceived of the study, and participated in its design and coordination and revised the manuscript. All authors read and approved the final manuscript.

## Supplementary Material

Additional file 1 **Results of variable testing for the three cases during the following-up period**. The data provided represent the results of variable testing for the three cases during the following-up period Click here for file
