# Supplementary material for: Current antibody-based immunoassay algorithm failed to confirm three late-stage AIDS cases in China: case report
Source: Virol J. 2010 Mar 15;7:58. doi: 10.1186/1743-422X-7-58 (PMC2850901; doi:10.1186/1743-422X-7-58)
Supplement: Additional file 1 — Results of variable testing for the three cases during the following-up period. The data provided represent the results of variable testing for the three cases during the following-up period [file 1743-422X-7-58-S1.DOC]

**Table 1. Results of variable testing for the three cases during the following-up period**

| Case | Date | The result of value of variable | | | | | | | | | | | | | |
| --- | --- | --- | --- | --- | --- | --- | --- | --- | --- | --- | --- | --- | --- | --- | --- |
|  |  | ELISA screening assays | | | | WB | CD4  （count/μl） | VL | Clinical parameters | | | | | | |
|  |  | ^a^ELISA1 (S/CO) | ^b^ELISA2  (S/CO) | PA | RT |  |  |  | Fungous  infection | Herpes | HBV | HCV | syphilis | CMV | Karpos’s sarcoma |
| A | June 10, 2005 | ND | ND | + | + | ID（p24） | 17 | ND | + | - | - | - | - | - | - |
|  | January 19, 2006 | ND | ND | - | - | ID（p24） | 9 | 10^6^ | + | - | - | - | - | ND | - |
|  | May 18, 2006 | +(3.67) | +(1.17) | ND | ND | ID（gp160 p24p17） | 95 | <10^3^ | - | - | - | - | - | ND | - |
|  | December 7, 2006 | +(2.12) | +(5.40) | ND | ND | ID（gp160 p24p17） | 238 | <50 | - | - | - | - | - | ND | - |
|  | March 10, 2007 | ND | ND | + | + | ID（gp160 p24p17） | 275 | <50 | - | - | - | - | - | ND | - |
|  | June 5, 2008 | +(15.09) | +(9.82) | ND | ND | +（gp160gp120  p24p17） | 323 | <50 | - | - | - | - | - | ND | - |
| B | January 8, 2009 | +(14.22) | +(5.27) | ND | ND | ID（gp160gp120） | 4 | ND | + | - | + | - | - | - | - |
|  | January 22, 2009 | +(16.82) | +(2.90) | ND | ND | ID（gp160gp120  gp41p66） | 1 | ND | + | - | + | - | - | ND | - |
|  | March 30, 2009 | +(8.37) | +(6.41) | ND | ND | ID(gp160) | 11 | 5×10^3^ | - | - | + | - | - | + | - |
|  | April 30, 2009 | +(15.80) | +(7.11) | ND | ND | ID(gp160) | 29 | ND | - | - | + | - | - | + | - |
|  | May 27, 2009 | +(14.49) | +(3.28) | ND | ND | ID(gp160gp120) | 42 | ND | - | - | + | - | - | + | - |
|  | August 17, 2009 | +(12.93) | +(8.01) | ND | ND | +（gp160gp120  p24） | 63 | ND | - | - | + | - | - | + | - |
| C | October 8, 2008 | ND | ND | ND | ND | ND | ND | ND | ND | + | ND | ND | ND | ND | ND |
|  | May 7, 2009 | +(17.92) | +(17.23) | ND | ND | gp160p24 | 35 | 10^6^ | - | + | - | - | - | - | - |
|  | August 13, 2009 | +(12.54) | +(23.74) | ND | ND | ID（gp160gp120） | 8 | ND | - | + | - | - | - | - | + |

^a^ELISA1: bioMérieux third generation, HIV1/2

^b^ELISA2: Beijing BGI-GBI third generation, HIV1/2

PA: partial assay, Nenan Sino-American Biotech Co., Ltd, HIV1/2

RT: rapid test, selenium labeled, Dainabot Co., Ltd, HIV1/2

WB: confirmation assays (western blot HIV1/2, HIV Blot 2.2 Genelabs Diagnostics, Singapore)

VL: viral load, viral RNA copies/ml (AMPLICOR HIV-1 MONITOR TEST version1.5. Roche, Germany)

+: Positive

-: Negative

ID: Indeterminate

ND: no data
